# Mitigating the Impact of Air Pollution on Human Health

**DOI:** 10.1021/acs.est.6c03505

**Published:** 2026-05-23

**Authors:** James J. Schauer

**Affiliations:** 5228 University of Wisconsin—Madison, Madison, Wisconsin 53706, United States

**Keywords:** air pollution, health effects, exposure, atmospheric particulate matter

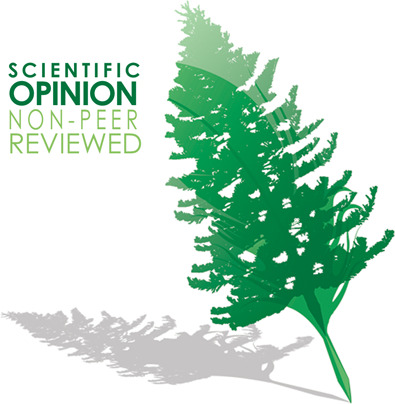

## Graphical abstract

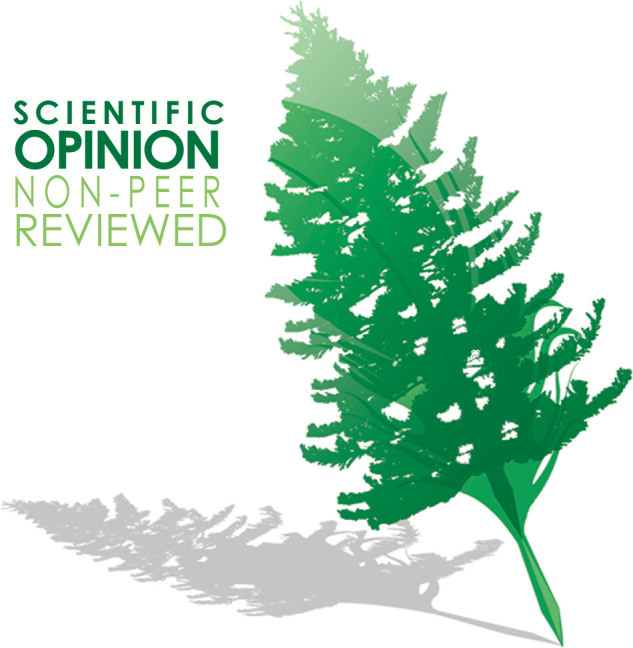

The biological mechanisms associated with air pollution health effects are remarkably different from the health effects resulting from exposures to chemicals in water, soil, and food. The inhalation of very small concentrations of air pollutants leads to localized oxidative stress and inflammation in the lung, as well as systemic changes in the human body resulting from cytokine and chemokine signaling to virtually all organs in the human body.^1^ The combination of localized impacts in the lung and systemic impacts across the human body results in significant air pollution health impacts, including respiratory disease, cardiovascular disease, neurological disease metabolic disorders, autoimmune disorders, cancer, and reproductive disease. Globally, ambient air pollution plus household air pollution is estimated to contribute to 7.9 million deaths per year, which is the second highest attributable risk factor for death after high blood pressure.^2^ Even in high-income countries, which generally have much lower air pollution levels than lower income countries, air pollution is estimated to cause about 450 000 death per year.^2^ These cumulative impacts of air pollution are dominated by atmospheric particulate matter and ozone.

Over the past several decades, significant progress has been made in advancing epidemiological studies to understand the associations of air pollutants and adverse health impacts.^3^ These advances have led to the promulgation of regulations and guidelines for air pollution limits in many locations around the world. In parallel, methods have been developed to understand the sources of air pollution that support mitigation strategies. In most high-income countries, efforts have been very successful in reducing air pollution concentrations but have not sufficiently reduced air pollution to fully mitigate the air pollution burden of disease. The current annual estimate of mortality from air pollution in high-income countries is in the range of 170 deaths per million people, which is very high compared to other environmental health risks.^2^ In regions of the world dominated by low-income and middle-income countries, air pollution levels are significantly higher and contribute to 1000–2000 deaths per million people annually.^2^

Historically, air pollution research studies have been bifurcated into two separate categories: (1) studies that examine the prevalence, sources, composition, and mitigation of air pollution and (2) studies that have examined the association of bulk properties of air pollution, such as particulate matter mass and ozone, with human disease. As a result, there is very limited information about the differential effects of exposures to different sources and components of air pollution.

Most modeling studies that seek to assess the impact of atmospheric particulate matter control programs and specific control strategies on human health inherently assume that there are no differential biological impacts of different sources and different components of particulate matter.^4^ Existing toxicological studies strongly suggest that this assumption is incorrect and different components of particulate matter and different sources of particulate matter dominate the biological effects of exposures to air pollution.^5^

It is worth noting that a parallel limitation exists in health effect studies that address photochemical oxidant pollution. Photochemical pollution contains ozone and numerous other chemical oxidants that are biologically active, but health studies typically use ozone as an exposure metric. Changes in emissions of photochemical oxidant precursors will change the distribution and relative amounts of photochemical oxidants in the atmosphere compared to ozone. The different mixtures of photochemical oxidants will have differential human health impacts.

Optimization of the design and implementation of air pollution control programs would greatly benefit from knowledge of the relative importance of air pollution sources and components on health effects. This information can be combined with traditional source apportionment studies that have been used to develop air pollution control programs. The integration would effectively replace current effects that focus on apportionment of air pollution concentrations with apportionment of air pollution health effects, which would better guide the development of air pollution control programs.

There are three areas of air pollution research that would greatly improve the ability to design and manage air pollution control programs that seek to reduce the impact of air pollution on human health.

(1) Implementation of toxicological and mechanistic studies that identity how different sources and components of air pollution impact the pathogenesis of the diseases that are impacted by air pollution sources. These studies will require an interdisciplinary approach to implement toxicological and epidemiological studies that can accurately quantify how exposures to different sources and components of air pollution impact the development and severity of specific diseases impacted by air pollution exposures.
(2) Apply the tools developed to understand how different components and sources impact the pathogenesis of disease to quantify how air pollution control technologies and atmospheric processing of air pollution change the health impact of air pollution emissions. Complete removal of all air pollution is not a realistic near-term goal for most urban areas due to the ubiquitous nature of air pollution. However, the development of strategies to maximize the reduction of the sources and components of air pollution that have the greatest impact on human health, considering both the contribution to pollutant mass and the relative biological impacts, will provide a more effective strategy to manage the health impact of air pollution.
(3) Given an understanding of the mechanisms of how specific sources and compounds of air pollution impact human disease, there are opportunities to explore medical interventions for susceptible populations to mitigate the impact of air pollution. The dominant burden of air pollution on human disease is on the young and the elderly,^2^ and opportunities to better protect these groups in circumstances where air pollution has not been properly managed could be beneficial to reducing the impacts of air pollution.
